# Examining the trade-offs of palm oil production and consumption from a sustainable diets perspective: lessons learned from Myanmar

**DOI:** 10.1017/S1368980021004353

**Published:** 2022-04

**Authors:** Shauna M Downs, Khristopher Nicholas, Kay Khine Linn, Jessica Fanzo

**Affiliations:** 1 Department of Urban-Global Public Health, Rutgers School of Public Health, One Riverfront Plaza, Suite 1020, Newark, NJ 07102, USA; 2 Carolina Population Center, University of North Carolina at Chapel Hill, Chapel Hill, NC, USA; 3 HelpAge International, Yangon, Myanmar; 4 Berman Institute of Bioethics, Nitze School of Advanced International Studies, and Bloomberg School of Public Health, Johns Hopkins University, Washington, DC, USA

**Keywords:** Palm oil, Myanmar, Value chain analysis, Consumer preferences

## Abstract

**Objective:**

The aim of this study was to examine the trade-offs related to the production and consumption of palm oil in Myanmar from a sustainable diets perspective.

**Design:**

We used an enhanced value chain analysis approach that included semi-structured interviews with key stakeholders; market analyses to assess edible oils in markets and focus groups as well as surveys with consumers to ascertain their perceptions and practices related to edible oils.

**Setting:**

Four settings in Myanmar (upper income urban; lower income urban; middle-income urban; lower income rural).

**Participants:**

Key stakeholders (*n* 12) from government, trade bodies and civil society organisations were included in the interviews. Women from each of the regions participated in four focus groups (*n* 32), and a convenience sample of male and female consumers participated in the surveys (*n* 362).

**Results:**

We found mistrust of the oil sector overall. Poor production practices, leading to low yields, limit the economic viability of oil palm production in Myanmar and contribute to negative environmental (e.g. deforestation) and social outcomes (e.g. land conflicts). Consumers demonstrated low preferences for palm oil as compared with traditional oils from a taste, health and transparency perspective; however, they indicated that its relative low cost led to its purchase over other oils.

**Conclusions:**

The Burmese example suggests that there may be limited benefits, and significant costs, of investing in palm oil production in regions where there are coordinating disincentives from a sustainable diets perspective. However, if oil palm cultivation is to continue, there are opportunities to improve its economic viability and environmental sustainability.

Sustainable diets encompass health, environmental, social and economic dimensions of the way in which food is produced, moves through the food system and is ultimately consumed^(1,2)^. Given the breadth of dimensions that relate to sustainable diets, they are prone to trade-offs among their different elements. Industrial crop systems, such as oil palm production, can intersect with sustainable diets in multiple ways^(3)^. Understanding the trade-offs within these systems is complicated and will continue to be a challenge in terms of promoting sustainable diets in the face of land competition as well as climate variability and change, particularly in low- and middle-income countries^(3)^.

Palm oil is the most widely consumed edible oil globally^(4)^. It makes up 35 % of the global edible oil supply^(5)^. As global demand for edible oils continues to rise, palm oil will likely play a prominent role in meeting demand^(5)^. It has been projected that oil palm production will need to increase by 25 million tons each year for the next decade to match rising global demand^(6)^. Currently, most palm oil (∼85 %) is produced in Indonesia and Malaysia^(7)^. However, due to the cultivation of increasingly less suitable lands and diminishing per hectare yields, these supply lines may be reaching peak production^(8)^. More significant future expansion of palm oil will likely occur in other parts of the world^(9,10)^.

As with most crops, there are economic, environmental, social and health trade-offs of producing and consuming palm oil^(10)^. Economic advantages of producing oil palm include its high yield compared with other edible oils which require substantially more land to produce the same amount of oil^(7,10,11)^, increased income and employment opportunities on farms, plantations and in processing mills and reduced poverty^(10)^. However, the oil palm boom has generated criticism related to the environmental and social consequences of its increased production^(10)^. Its production is associated with increased air pollution due to the burning of peat, land degradation, water pollution and significant losses in biodiversity associated with the clearing of forests^(10,12,13)^.

From a nutrition standpoint, palm oil contains a high proportion of saturated fat as compared with most other edible oils^(14,15)^. While red palm oil contains a high content of antioxidants, beta-carotene and vitamin E^(16)^, most of the palm oil consumed globally is refined, bleached and deodorised. Given that there is evidence to suggest that substituting palm oil for oils with more unsaturated fatty acids may be more healthful^(17,18)^, shifting consumption away from palm oil to unsaturated oils could improve diets and health outcomes. The WHO advises that ‘*unsaturated fats (e.g. found in fish, avocado, nuts, sunflower, canola and olive oils) are preferable to saturated fats (e.g. found in fatty meat, butter, palm and coconut oil, cream, cheese, ghee and lard)*’^(19)^. This messaging is consistent with food based dietary guidelines in nearly a third of countries globally^(20)^.

## Overview of Myanmar

Indonesia and Malaysia produce the majority of palm oil globally. However, other countries, including Myanmar, have begun producing palm oil in order to reduce their reliance on imports from a food sovereignty perspective^(21)^. While sesame and groundnut oils were traditionally used in Burmese cooking, palm oil import has increased dramatically in Myanmar since the 1990s^(22,23)^. Moreover, in the past two decades, Myanmar has incentivised investment in domestic palm oil production in the southern Tanintharyi region, an area of rich biodiversity and home to several ethnic minorities^(24–29)^. Tanintharyi’s palm oil plantation development and expansion has reduced forest cover by over 70 000 hectares from 2002–2014 alone^(30)^. The challenges introduced from this rapid change in land use due to oil palm development highlight the complex socio-ecological factors at play.

As Myanmar, as well as other countries globally, expands oil palm production, understanding the economic, health, social and sustainability trade-offs of these shifts, along with their drivers, will allow for the identification of more nuanced policy interventions for private sector innovation aimed at creating synergies among health, environmental and economic goals. Using an enhanced value chain analysis, this study examines the incentives and disincentives for the sustainable production and consumption of palm oil by conducting a case study of Myanmar and identifies potential intervenable points in the value chain to improve its economic viability, sustainability and health implications.

## Methods

### Conceptual underpinning and methodological approach

The conceptual underpinning of our approach centered on the various elements of sustainable diets. Given the increased global attention being placed on sustainable diets, we used an enhanced form of value chain analysis that incorporated both health and sustainability considerations. We adopted a broad view of sustainability that includes environmental (e.g. greenhouse gas emissions, water use, deforestation, etc.) and socio-cultural (e.g. consumer preferences, equity issues, labor conditions, land tenure, etc.) considerations based on a previously published sustainable diets policy analysis framework^(31)^. Supplemental Figure 1 provides an overview of the dimensions of sustainable diets considered in this manuscript.

Our methodological approach was informed by a combination of several value chain analysis methods (e.g. consumption-oriented food supply chain analysis, political economy analysis, etc.)^(32)^, with a particular emphasis on the value chains for nutrition framework developed by Gelli *et al*.^(33)^ for identifying, designing and evaluating interventions. It was conducted in four main steps: (1) analysing the macro-level food system context as it relates to edible oils in Myanmar; (2) characterising palm oil consumption patterns and identifying constraints to consuming alternative oils; (3) characterising the health, environmental and social properties of oil palm production and consumption and (4) identifying intervenable points in the value chain to strengthen the edible oil sector in an effort to support sustainable diets. A combination of document analysis, semi-structured interviews with key stakeholders, focus groups with consumers and market and consumer surveys were conducted. Data collection was conducted between June-August 2017. This study was approved by the Johns Hopkins University institutional review board. Local study partners included a conservation non-governmental organisation and a survey research organisation. All study participants provided informed oral consent to participate in the study.

### Semi-structured interviews and document analysis

The lead author conducted semi-structured interviews with key stakeholders (*n* 12) who had expertise related to Myanmar’s palm oil sector from government (agriculture, nutrition and health), civil society organisations and non-governmental organisations (agriculture, sustainability and consumer rights organisations) and trade bodies (oil processing, retail and trade and food science and technology). Preliminary document analysis, a formative field visit by the authors (S.M.D. and J.F.) where they met and informally interviewed several stakeholders (>15) from United Nations agencies, non-governmental organisations, civil society organisations, academia, etc., and local non-governmental organisation partners who were directly engaged in the palm oil sector helped to identify initial stakeholders. Additional stakeholders were identified through snowball sampling. We continued to identify additional interviewees until the point that we were not obtaining any new information from the interviews that we conducted. We selected interviewees based on their knowledge of the sector and their expertise related to different steps of the supply chain. While we attempted to interview firms directly involved in the production and processing of palm, we were unable to secure interviews with them. The interview guide was based on the different steps in the palm oil value chain and was tailored to the individuals’ areas of expertise. Supplemental Table 1 provides an overview of the interview guide, which was subsequently tailored to the value chain actor being interviewed. All but three of the interviews were recorded and transcribed verbatim. For those interviews that were not recorded, detailed notes were taken during the interview. We complemented the interviews with document analysis of policy documents, reports on the palm oil sector and with existing data from the FAO on edible oil imports and exports.

### Focus groups with consumers

We conducted consumer focus groups in four settings in Myanmar (upper-income urban township of Yangon; lower income urban township of Yangon; middle-income urban township in the Southern Myanmar town of Dawei; lower income rural village in the country’s dry zone of Magway) to better understand consumers’ consumption patterns, preferences and their decision making related to edible oils. Additional details regarding the focus groups, market and consumer surveys have been previously published^(34)^. In each of the study settings, our local partner would purposively select focus group participants with the assistance of community leaders to ensure variation in terms of age of participants^(34)^. In the case of the upper-income urban township in Yangon, additional sampling techniques were needed given the difficulty in identifying potential participants. In this case, women were identified through the existing networks of our local partner. Women were asked whether they made food purchasing decisions within their household prior to participating in the focus group – all women approached to participate in the focus group indicated that they did. A semi-structured focus group guide that included questions related to oil consumption, purchase and preferences was used (see online Supplemental Table 2). Each focus group contained eight women participants (*n* total 32) (age range 21–61 years; mean 40 years) who made food purchasing decisions within their household. Focus group participants received an incentive for participation (20 000 Burmese Kyat; equivalent to approximately US$15) based on recommendations from local partners. A trained Burmese facilitator guided the focus group discussions. In addition to the focus group facilitator, an additional researcher took detailed notes of the focus group discussions. The focus group discussions were recorded, transcribed verbatim and translated into English for data analysis.

### Market surveys

We conducted market surveys at five markets in each of the four study settings. A total of twenty market surveys were conducted in the markets that focus group participants mentioned they frequented the most. We obtained the price of each oil (sesame, groundnut, palm, sunflower, vegetable and soybean) from three different vendors in each market. However, in some cases, specific oils were not available in the market. We also asked whether the oil was sourced domestically or was imported and examined whether or not they contained labels.

### Consumer surveys

A total of 400 consumer surveys were completed (with men and women) at the twenty markets surveyed (see online Supplemental Table 3 for overview of socio-demographic characteristics). However, we removed thirty-eight survey respondents who did not reside in the study settings (total sample *n* 362). Two enumerators were stationed at the markets and asked consumers over the age of 18 years to participate in the study as they passed by, which is a typical approach in street intercept surveys^(35)^. Intercept surveys were conducted outside of the markets with consumers who were attending the market. Participants received a nominal incentive for participation (1500 Burmese Kyat; equivalent to approximately US$1). Consumer surveys included questions related to oil preferences and consumption, the primary oils used for cooking at home, the reason for using that oil (i.e. taste, price, availability, convenience, health or other) and which oil they considered the cheapest, the tastiest and the healthiest.

### Analysis

All data sources were combined and examined at each step of the palm oil value chain – inputs; production; processing; storage, distribution and trade; retail; and consumption – to examine the incentives and disincentives related to sustainable palm oil production and consumption. The interview transcripts, focus group transcripts, detailed notes and documents were open-coded and organised based on key themes related to the incentives and disincentives for palm oil production and consumption throughout the steps of the value chain using NVivo (version 11.4.2). We then triangulated these data from the quantitative data obtained from the consumer and market surveys. To identify points for intervening throughout the supply chain, we first determined where the bottlenecks in the current supply chains were, and subsequently identified potential interventions to address them based on the interviews, focus groups and market analyses. References are provided within the results section to denote when documents, rather than interviews, focus groups or surveys, are the source of the information described. Interview and focus group quotes are used throughout the results section to illustrate key themes derived from the qualitative data.

In order to examine incentives and disincentives for palm oil consumption at the consumer level, we used descriptive statistics to describe preferences for edible oils and their perceived characteristics. Differences in edible oil preferences based on study setting were examined using chi-squared tests. In order to assess market level incentives and disincentives for palm oil purchase, we used descriptive statistics to examine their availability and price. All quantitative analyses were conducted using SPSS (version 24). A *P*-value of < 0·05 was used to denote statistical significance.

## Results

### Overview of palm oil value chain

**Fig. 1:**
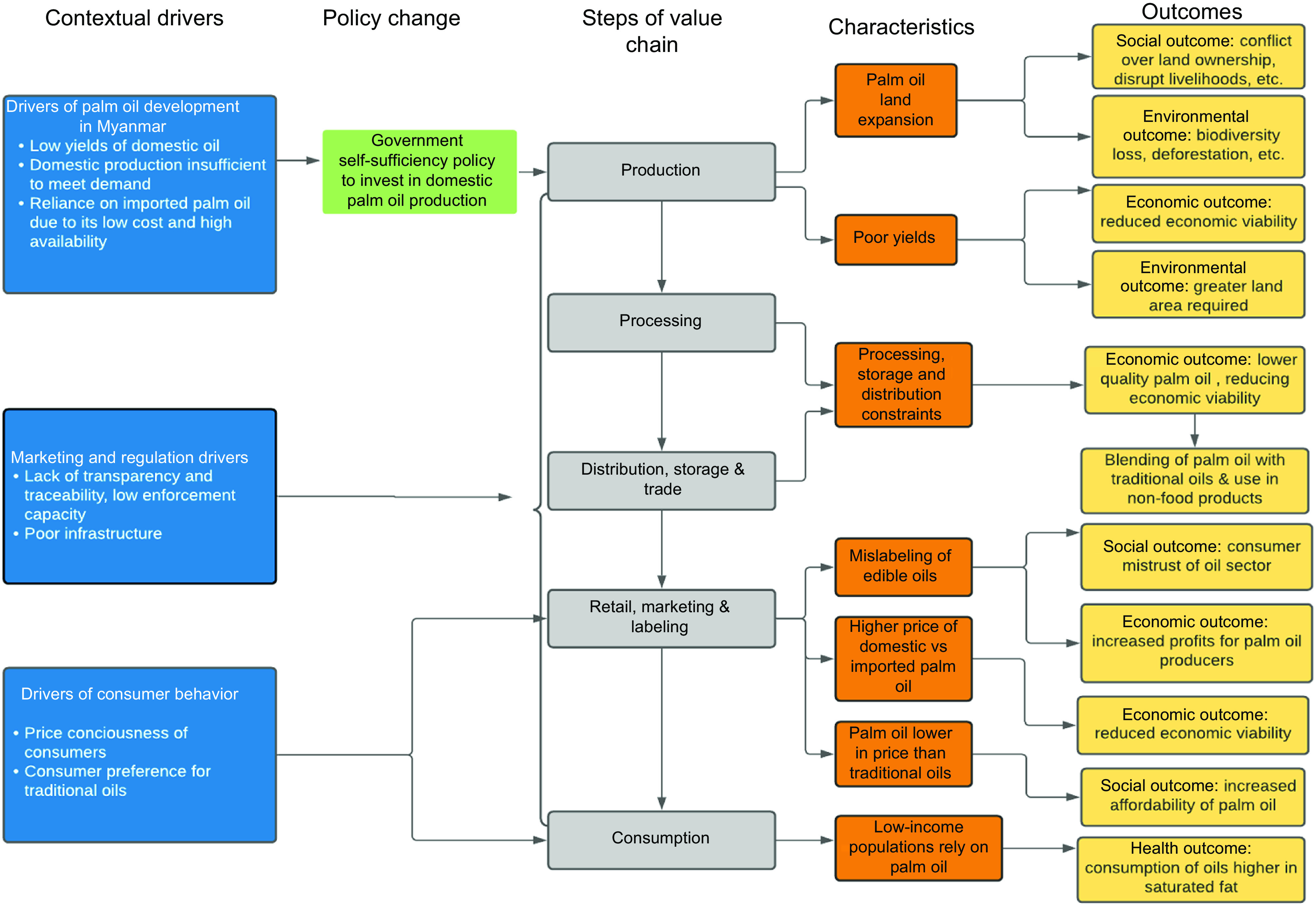
An overview of the edible oil sector in Myanmar

Figure 1 provides an overview of the drivers of the palm oil sector in Myanmar and its consequences, based on our study findings. There were several cross-cutting drivers related to the edible oil sector in Myanmar that have consequences for the palm oil supply chain. Key informant interviewees described a lack of transparency, including as it relates to the availability of palm oil production statistics in the country, as well as traceability throughout edible oils supply chains, which makes it difficult for consumers to know what they are purchasing and the source. In addition, there continues to be governance challenges (including corruption) and political instability in the country. Along with these challenges comes a lack of enforcement capacity to improve transparency in the edible oil supply. Lastly, a lack of strong infrastructure (e.g. roads) plagues the edible oil sector, as well as the agricultural sector more broadly, at each step of the supply chain.

In recognising its reliance on imported palm oil, the Myanmar Government adopted a self-sufficiency policy in the early 2000s. The self-sufficiency policy centered on the Tanintharyi region of Southern Myanmar becoming the ‘oil bowl’ of the country by investing in palm oil production. However, palm oil production in the region has had limited success due to supply chain constraints that are unique to Myanmar, including suboptimal production practices and low yields as compared with neighbouring palm oil producing countries. Although there are a small number of companies that are generating profits, in most cases, the yields and quality of the palm oil produced are low. Based on focus group discussions and market surveys, consumers prefer the oils that have traditionally been produced in the country (groundnut and sesame), yet the productivity of those oils is low given numerous constraints (e.g. lack of irrigation, low quality seed, antiquated machinery).

Table 1 provides an overview of the key challenges across the Burmese palm oil value chain that influence its production, the way it moves along the value chain, as well as its consumption and whether they have a positive, negative or neutral consequence on the economic viability of the sector, sustainability and human health. We describe the interrelationship between these opportunities and challenges and the trade-offs they create below.

**Table 1:** Overview of the key challenges across the palm oil value chain in Myanmar and their consequences

Steps of value chain	Key challenges	Description	Consequence		
			Economic viability	Sustainability	Human health
Production	Suboptimal agro-climatic conditions	Inconsistent rainfall Topography of land (e.g. hills) Soil acidity	↓	↓	–
	Suboptimal management practices	Inappropriate application of fertilizer Lack of financial capital to invest in plantations Lack of experience among plantation owners	↓	↓	–
	Lack of electricity	Electricity challenges create bottlenecks for refineries	↓	–	–
	Concessions to private sector to engage in palm oil production	Land concessions Subsidies on diesel, duty-free import of machinery, waiving of commercial tax	↑	↓	–
	Misuse of concessions	Clearing of land for logging rather than palm oil production	↑	↓	–
	Relatively low productivity levels	Yields lower in Myanmar compared with Indonesia and Malaysia Higher cost of production in Myanmar	↓	↓	–
	Land tenure conflict	Accusations of land grabbing and expansion of forest clearing beyond their permits by palm oil companies Difficulty for indigenous populations to provide documentation of their land rights Tensions between community members and palm oil companies Production in historical conflict areas	↑	↓	–
	Majority of labour force on plantation are migrant workers	Low quality living conditions for migrant workers Limited local employment opportunities	↑	–	↓
	Limited conservation efforts	Focus on sustainable palm oil production viewed as being premature RSPO certification deemed too difficult for most companies Limited knowledge related to conservation practices	↑	↓	–
Processing	Limited processing capacity	Insufficient processing facilities and the quality of those facilities were considered poor Few of the companies in Myanmar have their own processing plants, which means they rely on selling their fruit to larger companies to process them Limited refineries to process crude oil into refined oil Long distances and poor roads create barriers to refinement	↓	↓	–
	Blending of palm oil with other oils	Palm oil blended with other oils, such as groundnut or sesame, and being sold in markets without accurate labelling	↑	–	–
Distribution, storage and trade	Lack of traceability	Little is known about how much palm oil is produced in the country, or what happens to it once it has been produced. There are no official statistics regarding palm oil production in Myanmar			
	Reliance on palm oil imports	Insufficient production of domestically produced oils Relatively high cost of domestically produced foods	↑	–	↓
Retail	Low cost of palm oil	Domestically produced palm oil more costly than imports	↑	–	–
	Lack of enforcement of adulterated oils	Lack of capacity among the Myanmar FDA for monitoring and enforcement High cost of testing oils to ensure proper labelling seen as a barrier to scaling up enforcement to ensure that the oils sold are unadulterated and that labelling reflects the true contents of oil	↑	–	↓
Marketing & labelling	Inaccurate labelling of oils	Oils that were labelled as vegetable oils in the country were also predominantly palm oil	↑	–	–
	Lack of labels	Many oils, particularly domestically produced, lack labels Oils purchased as ‘loose’ in informal market	↑	–	–
Consumer	Consumers prefer traditional oils	Less trustworthiness of palm oil produced within Myanmar compared with imports Preference for oils that are liquid rather than solid (e.g. lower in saturated fat) Perceived low quality of palm oil Perception that palm oil is relatively less healthy Purchasing oil directly from rural villagers to ensure ‘pure’ oil	↓	–	↑
	Price consciousness of consumers	Consumers have limited purchasing power Lower quality relatively cheaper Blended oils cheaper than ‘pure’ oils	↓	–	↓

↓ Decrease in outcome; ↑ Increase in outcome.

### Domestic palm oil production

The government provided several incentives to companies to establish oil palm plantations in the Tanintharyi region; however, it has had limited success. It was suggested in the stakeholder interviews that some companies cleared the land to profit from logging rather than oil palm production. As one stakeholder said: ‘*But no need to grow…no need to grow palm oil. It’s very difficult and very hard work. Just using that kind of opportunity is much much better*’ (agronomist, palm oil expert). Although interviewees indicated that there are a small number of companies generating profits in Myanmar, in most cases, the yields and quality of the palm oil produced are low due to several reasons described in Table 1. Many of the companies that entered into the palm oil sector with government-provided land concessions did not have prior experience growing palm oil. This led to suboptimal production practices and, in some cases, limited investment to improve those practices. As one stakeholder from the agriculture sector stated: ‘*That’s why, even though they want to develop the project, but because of the lack of support, they can do nothing*.’

Interviewees indicated that while companies have grappled with production challenges related to increasing yields, they have also been dealing with the social and environmental issues associated with the palm oil sector in Myanmar. Land tenure is one of the main social challenges related to palm production in the area. There have been accusations of land grabbing by companies and expansion beyond permitted areas in some parts of the Tanintharyi region, creating tension between the palm oil companies and local communities. As one stakeholder from a sustainability non-government organization (NGO) said: ‘*Your basic human right to livelihood. You have the right to protect your environment. You have the right to have property and the plantations. You have the right to ask compensation. Since they have grabbed your land*.’

One potential benefit of increasing palm production in the area is employment creation. However, the majority of palm oil plantation laborers are migrant workers, many of whom live under poor conditions^(36)^. Interviewees indicated that the government ‘*don’t want to get involved in this issue*’ given the complexity and the difficulty in finding solutions.

In Myanmar, forests have been cleared to plant palm oil^(37,38)^. However, given the lack of knowledge related to appropriate conservation practices, there has been total clearing of forests: ‘*We just know how to grow and how to harvest. That’s all. But our people do not have enough experience for environmental conservation. And we don’t have enough knowledge. We have very little knowledge of as to how to conserve the environment. Even we don’t know what is high conservation value? We also don’t know about as to how to conserve the biodiversity*’ (agronomist, palm oil expert). To address the environmental concerns related to palm production, there has been a push towards ‘sustainable’ production practices as part of the Roundtable on Sustainable Palm Oil (RSPO). In Myanmar, interviewees indicated that it was premature to seriously consider sustainability. As one oilseed industry expert indicated: ‘*Your question is very advance. Our people cannot think that. They are just thinking for their finances*.’ There was a perceived trade-off between investing in more sustainable practices and ensuring the economic viability of the sector.

### Distribution, storage and trade constraints

One of the main challenges related to better understanding the distribution of palm oil in Myanmar is the lack of traceability. Little is known about how much palm oil is produced in the country, or what happens to it once it has been produced. One interviewee with knowledge of the sector estimated that approximately 40 % of the palm oil produced in the country was being sold as cooking oil and the other 60 % was being sold in processed foods and for industrial uses such as soap, candles, etc. The majority of the lower quality oil was being used for the latter. However, official statistics about the total production of palm oil and its uses do not exist.

**Fig. 2:**
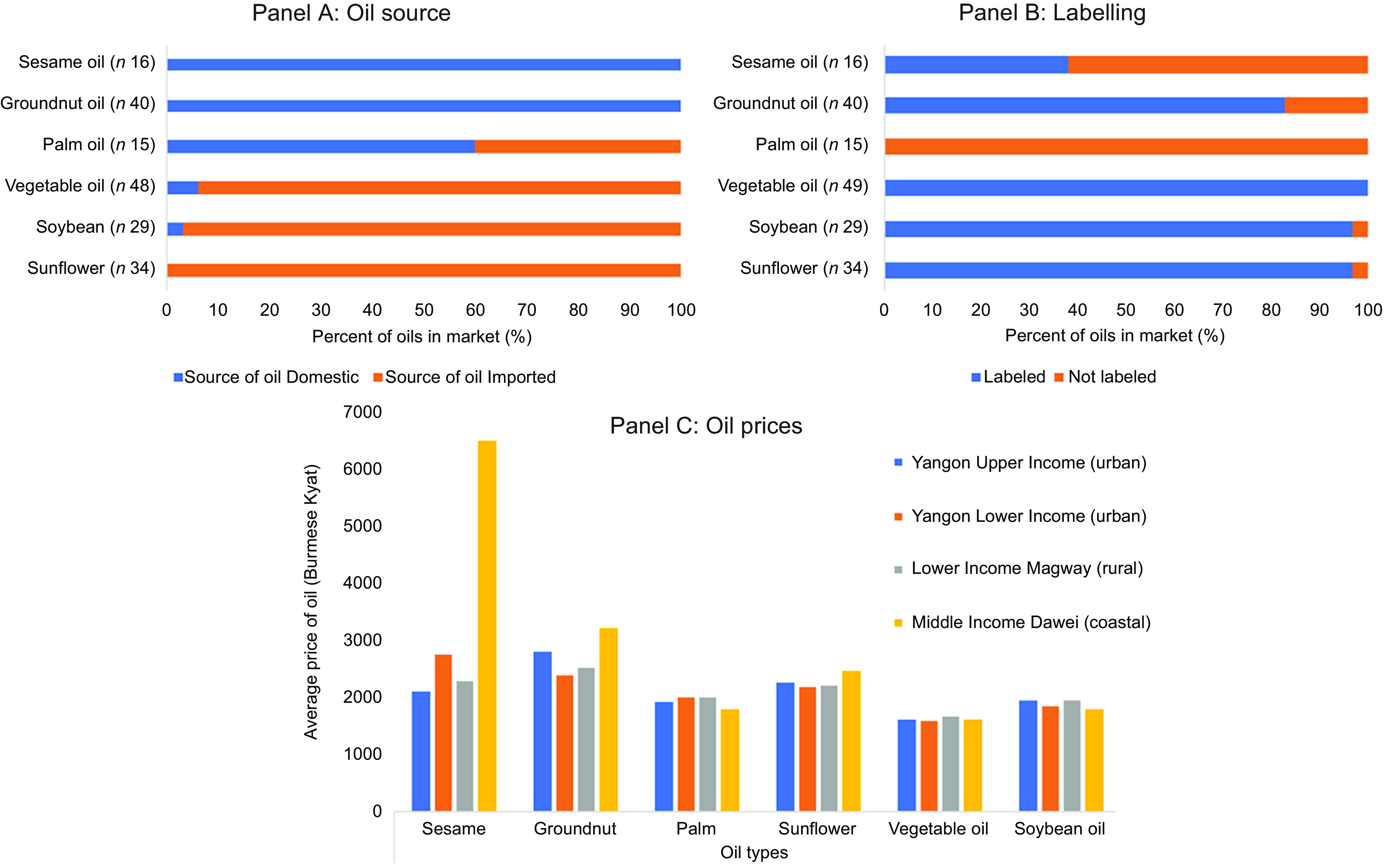
An overview of the source and use of labels among oils examined in markets (*n* 20) in four regions of Myanmar. Note: Vegetable oil refers to oil labelled as ‘vegetable’ oil without information about the specific oils included

At the same time that palm oil is being produced domestically, the country is importing large quantities of palm oil. Nearly all the edible oil imports in Myanmar consist of palm oil (see online Supplemental Fig. 2). Our analyses of the oils available in the markets showed that over half of the palm oil being sold was domestically produced, while the majority of ‘vegetable oil’ was imported (although it is likely that it mostly consisted of palm oil), and the traditional oils such as sesame and groundnut were all produced domestically (Fig. 2). In focus group discussions, consumers living in the Tanintharyi region of Myanmar, where palm oil is produced, indicated greater trustworthiness of the quality of imported oils from Thailand.

### Processing constraints

One of the main bottlenecks identified in the supply chain of domestically produced palm oil in Myanmar was processing capacity. There were insufficient processing facilities, and the quality of those facilities was considered poor. Fresh fruit bunches need to be processed within 24 h of being picked to create crude palm oil, which is subsequently refined further (i.e. secondary processing to refine, bleach and deodorise) prior to being consumed as cooking oil. Few of the companies in Myanmar have their own processing plants, which means they rely on selling their fruit to larger companies to process them. Moreover, there were few refineries in the Tanintharyi region for secondary processing of oils. These refineries were primarily based in the Yangon region rather than in the region in which the palm oil is grown. One interviewee indicated that this was due to more reliable electricity in the Yangon area as well as economic incentives from the government, including land concessions. However, the distance between Yangon and Tanintharyi was considered significant (∼1000 km). The long distance in addition to poor roads and infrastructure served as barriers to processing. Interviewees indicated that these challenges jeopardised the quality of the palm oil that was subsequently processed.

All interviewees indicated that palm oil was being blended with other oils, such as groundnut or sesame, and being sold in markets without accurate labelling. As one interviewee from a sustainability NGO stated: ‘*They are calling it groundnut when its groundnut mixed with palm oil*.’ Interviewees indicated that ‘*Because of this technique they can get the profit*’ (agronomist, palm oil expert). Interviewees stated that oils that were labeled as vegetable oils in the country were also predominantly palm oil. They also acknowledged that there was little disincentive for blending of oils without properly labelling them, given the lack of enforcement and insignificant fines.

### Retail, promotion and labelling constraints

The reliance on informal markets has made it easier to sell blended edible oils. A lack of capacity among the Myanmar FDA for monitoring and enforcement was also identified through interviews. The high cost of testing oils to ensure accurate labelling was also seen as a barrier to scaling up enforcement to ensure that the oils sold are unadulterated and that labelling reflects the true contents of oil.

Many of the oils sold in Myanmar were unbranded, lacking labels and packaging, including those that are imported. In the market analyses, we found that none of the palm oil available within the markets we examined contained labels, while all, or nearly all, of the vegetable, soybean and sunflower oils, the majority of which were imported, contained branded labels (Fig. 2). Moreover, the oil purchased at smaller retailers was still often sold ‘loose’ (i.e. not in a container). As one interviewee from a food safety NGO said: ‘*Somebody come to the small shop, they bring their own container and then the seller put in that, they carry it away and they use*.’

### Consumption of palm oil

**Fig. 3:**
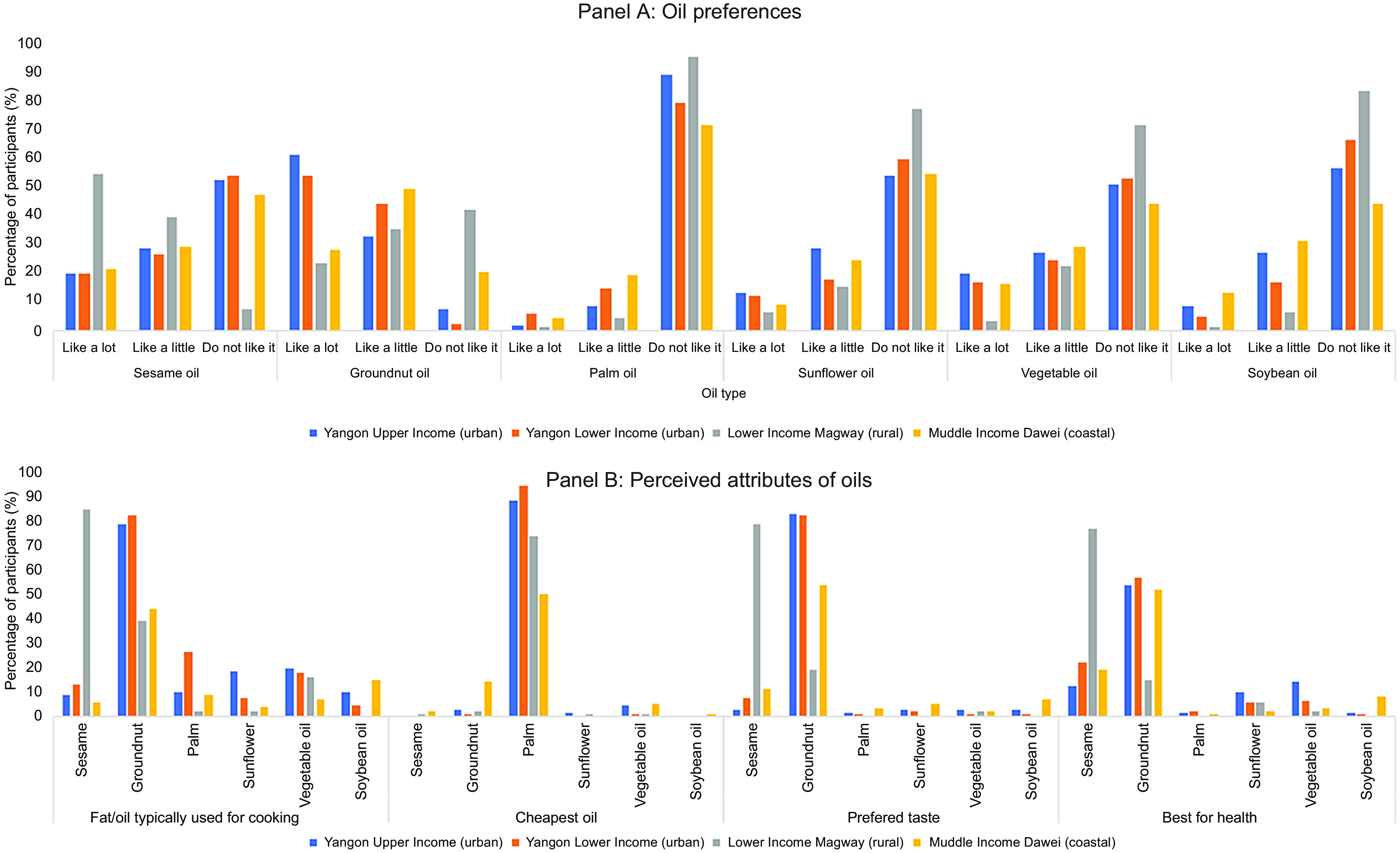
An overview of edible oil preferences and perceived attributes in four communities in Myanmar. Significant differences in edible oil preferences and oils typically used using Chi-squared test (*P* < 0·01)

Figure 3 provides an overview of consumers’ preferences for different oils based on the consumer surveys. There were significant differences in the taste preferences for different oils based on study setting, with both upper- and lower-income urban, as well as those living in the Tanintharyi region, preferring groundnut oil while rural consumers, who lived in the sesame growing region, preferred sesame oil (*P* < 0 001; Panel A). The oils typically used for cooking also varied significantly among regions (*P* < 0 001), with the highest proportion of participants using palm oil as their cooking oil in the lower income neighbourhood in Yangon (Panel B). A similar pattern was found for the perceived healthfulness of the different oils, where sesame was perceived to be the healthiest oil among rural consumers, while groundnut was considered the healthiest among consumers in the other study settings. With the exception of palm oil and vegetable oil, health was the main factor influencing use of a given type of edible oil (see online Supplemental Fig. 3). Consumers most often reported cooking with palm oil because of its price, availability and convenience, with very few consumers choosing palm oil for its healthfulness.

We found a considerable amount of mistrust in the entire edible oil sector. As an interviewee from a consumer organisation said: ‘*We all are worried whether we are having a safe oil or not*.’ For this reason, oils that were labelled as having ‘FDA approval’ were seen as being more trustworthy. Based on focus group discussions, some consumers attributed the mixing of oils without appropriate labelling of the contents as ‘*occurring because of the greed*’ of companies (Yangon, upper-income). The trade-off between quality and price was also described in the consumer focus group discussions, particularly among lower income participants. As one focus group participant from a rural low-income village said: ‘*Mixed oil is not good for health as groundnut oil and sesame oil. I can afford only the relatively cheap oil and so I have to use less expensive mixed oil although its taste is not so good. I consume mixed oil only because of its price and my income*.’

### Entry points for interventions to improve sustainability of edible oils

Based on the findings of the interviews, document analysis, market surveys, consumer surveys as well as focus group discussions, Table 2 provides an overview of potential points for intervening throughout the supply chain and their consequences regarding the sector’s economic viability, sustainability (including environmental and socio-cultural considerations) and human health. Many of the levers for intervening in the food supply apply to both palm oil as well as traditional oils produced within the country. Importantly, many trade-offs were identified as it relates to intervening to support increased palm oil production within Myanmar demonstrating the potential pitfalls of continuing to invest in this sector. However, given that Myanmar has shown no indication of easing oil palm cultivation, bolstering sustainability moving forward is imperative not only for the Burmese market but because of how interconnected health, environment and trade are with palm oil.

**Table 2:** An overview of entry points to improve the sustainable production and consumption of oils in Myanmar

Steps of value chain	Points for intervening in supply chain	Applies to traditional oils	Applies to palm oil production	Potential consequences			Potential trade-offs and synergies
				Economic viability	Sustainability	Human health	
Inputs and agricultural production	Increased access to credit	x	x	↑	↕	↕	Improved access and use of inputs could increase economic viability because of potential increase in yields, while also putting additional stress on the environment if not done in a sustainable way. Improved inputs could lead to palm oil expansion leading to negative social and environmental outcomes. Increased palm oil availability and affordability may reduce consumption of more healthful traditional oil (substitution effect).
	Investment in R&D	x	x				
	Increased access to irrigation	x	x				
	Improved fertilizer application	x	x				
	Access to improved farming machinery and technology	x	x				
	Stronger land tenure laws (including enforcement)	x	x	↓	↑	–	Customary land rights of local communities would be more sustainable in terms of protecting their livelihoods, enabling smallholder production, etc. Protecting customary land rights of local communities could reduce economic viability of palm oil sector.
	Improved knowledge transfer of best practices (including feasible conservation practices) for producing palm oil efficiently within the context of the agro-climatic conditions in the Tanintharyi region		x	↑	↑	↓	Improved knowledge transfer could increase both economic viability and sustainability of oil palm production through increased ecosystem services. Emphasis on conservation practices could increase labour requirements, which has the potential to generate more employment but also to further reduce economic viability of oil palm production.
	Limiting further palm oil production expansion		x	↓	↑	↑	Limiting further expansion of palm oil could reduce the economic viability of the sector but lead to improvements in terms of sustainability and health outcomes.
Primary processing	Investment/upgrading of processing facilities for small-scale oil producers	x	x	↑	↑	↑	This would increase the economic viability of the broader edible oil sector and increase sustainability through improved efficiency in terms of the proportion of oil extracted from the seed, fruit, nut, etc.
	Incentives for investment in improved processing technology	x	x				
	Increasing FDA certification of oil mills	x	x	↓	–	↑	Increased FDA presence could reduce blending of oils making palm oil less economically viable given that current blending practices without accurate labelling allow manufacturers to sell palm oil blended with traditional oils at a higher price. However, could improve the quality of product that reaches consumer with the potential to improve health outcomes.
Distribution, storage and trade	Improvements in infrastructure (e.g. roads, electricity, etc.)	x	x	↑	↑	↕	Improved infrastructure would increase economic viability and sustainability of edible oils due to products being moved more easily and quickly, with less waste. Could increase oil availability leading to variable health outcomes if consumption patterns shift.
Retail	Improved labelling of oils (stronger regulation, clear indication of breakdown of blended oils, etc.)	x	x	↓		↑	Improved transparency could reduce economic viability of firms that are mislabelling products and enable consumers to make more informed decisions.
	Improved enforcement of oil supply	x	x	↓		↑	•
Marketing	FDA certification logos	x	x	↓		↑	•

Arrows represent the direction of relationship between point of intervening and outcome.

↓ Decrease in outcome; ↑ Increase in outcome; ↕ variable (increase or decrease) in outcome.

## Discussion

Although there are often trade-offs from a sustainable diets perspective of increasing palm production and consumption, we found several coordinating disincentives for its production and consumption in Myanmar. Namely, relatively low productivity levels hinder the sector’s economic viability while consumers report low preferences for its consumption as compared with traditional oils. While our analysis suggests that diverting investment from palm oil to traditional oils in Myanmar may be warranted, we also highlight how the palm oil sector could be strengthened given that it is expected that oil palm cultivation will expand to regions around the world with suboptimal climatic conditions^(10)^. For those countries, there are lessons to be learned from Myanmar’s experience to better support sustainable diets.

### Economic and environmental considerations

Countries such as Myanmar that invest in domestic palm oil production will need to identify ways of increasing the economic viability of the sector while weighing the environmental, social and health costs associated with its production and consumption. Although a small number of companies in Myanmar are profiting from palm oil production, many of them are not, and the sector is not as economically viable as in Indonesia and Malaysia where the palm oil industry has led to large-scale economic growth^(10,39)^. However, for those companies where palm oil production is profitable, there is a trade-off between profitability and investing in more environmentally sustainable practices. The trade-off between producing sustainably and profitability is also experienced in other palm oil growing countries^(40,41)^. In Thailand, although little of the palm oil produced is RSPO certified, it is most often produced by smallholder farmers, rather than large plantations, and cultivation has been primarily in areas formerly used for producing other crops^(42)^. This has led to less deforestation associated with oil palm production in Thailand as compared with other palm producing countries^(42)^. Countries that expand oil palm production in the future could adopt an approach similar to Thailand which can help to address some of the environmental and social (e.g. land conflict) trade-offs related to oil palm expansion.

### Social considerations

The palm oil sector in Myanmar is plagued by issues related to the land rights of indigenous communities from the region, who often do not have documentation of their land holdings. This has also been observed in other palm oil producing regions globally^(10,43)^. Interviewees in our study indicated that given the complexity of this issue, the government was reticent to get involved. However, strong community engagement and governance structures are necessary in this context. One potential option for strengthening community engagement, alongside increased transparency with regard to land rights, is to invest in the smallholder production of palm oil, which has been done in Thailand^(44)^. In 2013, the Government of Indonesia recognised the constitutional rights of indigenous peoples to their lands and territories, including their collective rights over customary forests^(45,46)^. As countries move forward with palm production, these rights should be protected from the onset of oil palm expansion. In Myanmar, it will be unlikely that the palm oil sector will be able to gain the trust of the public without better engagement with local communities.

### Consumer considerations

Perhaps the most significant trade-off related to palm oil being weighed is for consumers and their health. We found that consumers perceived palm oil as being unhealthy as compared with other oils. While there is some evidence to suggest negative health outcomes associated with palm oil consumption from a nutrition perspective, the evidence is somewhat mixed^(47,48)^. Despite the lack of convincing evidence linking palm oil consumption with negative health outcomes, our findings suggest that consumers have a strong dislike of palm oil. We found that consumers’ preferences were more aligned with traditional oils as compared with palm oil but that the cost of those oils was often prohibitive. This is aligned with previous research that has shown food prices to influence diets among low-income population in Myanmar^(49)^. By improving productivity of these oils and strengthening processing and distribution, their affordability could increase. Given rapidly Westernising diets and homogeneity induced by industrial monoculture agricultural products like palm oil, supporting a return to traditional oil consumption may prove beneficial in other future palm oil markets as well.

### The need for better supply chain governance

Supply chain governance needs to be strengthened in countries such as Myanmar who pursue oil palm expansion. Currently, there is little transparency in the palm oil supply chains in Myanmar as it relates to both imported and domestically produced oils. It is likely that this lack of transparency will intensify with the current political instability within the country and the recent shift away from democracy. Given the number of departments and ministries involved in the food supply chain, ensuring a more cohesive approach to monitoring and enforcement of the sector is important. Historically, the FDA has not had sufficient capacity to ensure the quality of the edible oil supply in the country, as is evident in other countries^(50)^. Nevertheless, more recently, the FDA has signalled that they will increase surveillance at borders, and they are building laboratory facilities in order to be able to test foods^(51)^. Building the technical and human resource capacity of the FDA is necessary but needs to be coupled with strong governance aimed at increasing transparency along the entire supply chain. This will also help to address consumer concerns related to the trustworthiness of edible oils. With strong labelling regulation as well as monitoring and enforcement of that regulation, trustworthiness of the edible oil sector could be improved^(52)^.

### The need for investment by both the private sector and government

As with improved governance, if countries such as Myanmar expand palm oil production, increased investment in the sector is required. Given the low productivity levels of palm oil in Myanmar as compared with other countries, investment by the private sector is needed in order to adopt improved production strategies and technologies. This will likely be true in other countries that pursue oil palm expansion in less favourable climatic conditions. Access to credit and technology will be needed in these settings^(10)^, particularly for smallholder producers. At the same time, investment in R&D by both the private sector and the government could help improve production practices of palm oil as well as traditional oils. The private sector also needs to invest in increased storage and processing facilities to reduce bottlenecks in the distribution of palm oil once the fresh fruit bunches have been picked, given the time sensitivity. Investment in road infrastructure as well as providing incentives for upgrading processing facilities for traditional oils is also necessary to streamline their supply chains.

### Limitations

This study had several limitations. We were limited by a relatively small sample of key stakeholders. While we intended to conduct interviews with stakeholders representing each of the steps of the value chain, we were unable to secure interviews with palm oil producers, indigenous persons being displaced by oil palm production as well as other key groups. Our study was also limited by our lack of analysis of samples taken from oils sold in markets to triangulate data from interviews and market surveys. Lastly, we were also limited by our lack of consumption data.

## Conclusions

This value chain analysis proposes four key findings. First, given high cost of production and low oil palm yields, the economic viability of Myanmar’s palm oil industry is uncertain. Second, the environmental and social costs of industrial palm oil expansion are significant and settings such as Myanmar’s biodiverse and ethnically diverse Tanintharyi region are especially vulnerable. Third, large gaps in governance and monitoring of Myanmar’s edible oil sector contribute to data scarcity and misaligned incentives that make it extremely difficult to identify supply chain bottlenecks. Lastly, consumers have expressed strong preferences for traditional oils such as groundnut and sesame, casting into doubt the efficacy of strengthening palm oil supply chains *v*. improving the affordability and consumption of healthier, traditional oils. There are several lessons that can be learned from Myanmar’s experience with oil palm expansion that may be applicable to other countries that begin to expand production while supporting sustainable diets, including engaging with smallholder producers, investment in R&D to ensure that production practices are adapted to local climatic conditions, educating producers about conservation practices before they begin planting and strengthening the governance, transparency and trustworthiness of edible oil supply chains. Lastly, countries investing in edible oil production should consider health properties and ensure that there is synergy between nutrition and health guidance (e.g. food-based dietary guidelines) and production policies.

## Supplementary material

For supplementary material accompanying this paper visit https://doi.org/10.1017/S1368980021004353. click here to view supplementary material
