# Supplementary material for: Examining the trade-offs of palm oil production and consumption from a sustainable diets perspective: lessons learned from Myanmar
Source: Public Health Nutr. 2021 Nov 15;25(4):964–76. doi: 10.1017/S1368980021004353 (PMC9991760; doi:10.1017/S1368980021004353)
Supplement: Supplementary file 1 [file S1368980021004353sup001.docx]

**Supplemental Figure 1. An overview of a sustainable diets framework (adapted from Downs et al., 2017).**

**Supplemental Table 1. Semi-structured interview guide with key stakeholders in edible oil sector**

| **Overarching topic** | **Questions** |
| --- | --- |
| Oil production | - What are the existing edible oil policies in the country?   - How have these changed over time? - Why has palm oil production been promoted in the Tanintharyi region? - What are the existing incentives (policy, economic or social) for palm oil production? How do they compare to other domestically produced oils?   - How has increased palm oil production affected the surrounding communities? - What are the challenges associated with palm oil production in the country?   - How can they be overcome? - How have issues of sustainability been addressed in edible oil related policies?   - Which actions has the government taken to address sustainability?   - Which actions has the private sector taken to address sustainability? - How has palm oil production affected land use in the Tanintharyi region? - What are the barriers for producing edible oils?   - Palm oil   - Sesame oil   - Groundnut oil - How profitable is edible oil production?   - Palm oil   - Sesame oil   - Groundnut oil |
| Value chain | - What are the barriers to getting oils from producers to consumers?   - Palm oil   - Sesame oil   - Groundnut oil - How are oils distributed within the country? - What are the bottlenecks in the supply chain?   - Palm oil   - Sesame oil   - Groundnut oil - How efficient is oil processing for the different oils? Are there any by-products?   - Palm oil   - Sesame oil   - Groundnut oil - Where is the most oil wasted in oilseed value chains (i.e., from production, processing, distribution, retail to consumer)?   - Palm oil   - Sesame oil   - Groundnut oil |
| Consumer | - Which oils are most widely consumed in the country? Why?   - Probe: consumer preferences - What are the sociocultural norms related to oil consumption? |

**Supplemental Table 2. Focus group discussion guide: Consumers in Myanmar**

| **Overarching topic** | **Questions** |
| --- | --- |
| Oil consumption | - Which oils do you (your household) usually or mainly consume? Why? How much do you consume per week / per month? - Why do you consume this type of oil? - Do you (your household) have any other types of oil that you consume? Why? - Which oils do you think other people usually consume? If the type(s) of oil is different, why do you think they prefer that type of oil? - If you have a choice, [not worrying about the cost or accessibility] do you have another type of oil that you would want to consume, other than mentioned above? Why do you say so? - How do you compare different oils in availability and prices in rural and urban markets? - How do you compare different oils in availability and prices changed over time? |
| Shopping patterns | - Where do you usually shop for food and oil? - How often do you shop there? - How do you get to this outlet? - What motivates you to shop at this outlet? - How much time does it take to get there? [Probe: Does this change in the rainy season?] - How do consumption of oils contribute to your diet? [Do you think you eat more oil compared to other people? Do you think you eat a healthy amount?] Please explain. |
| Changes over time | - How have the food outlets that you have access to changed over time? - How have these changes influenced your diet over time? - How have your consumption patterns changed over time? - What are the barriers to accessing nutritious foods? - Which foods do you prefer to eat? Why? What are the barriers to accessing these foods? |


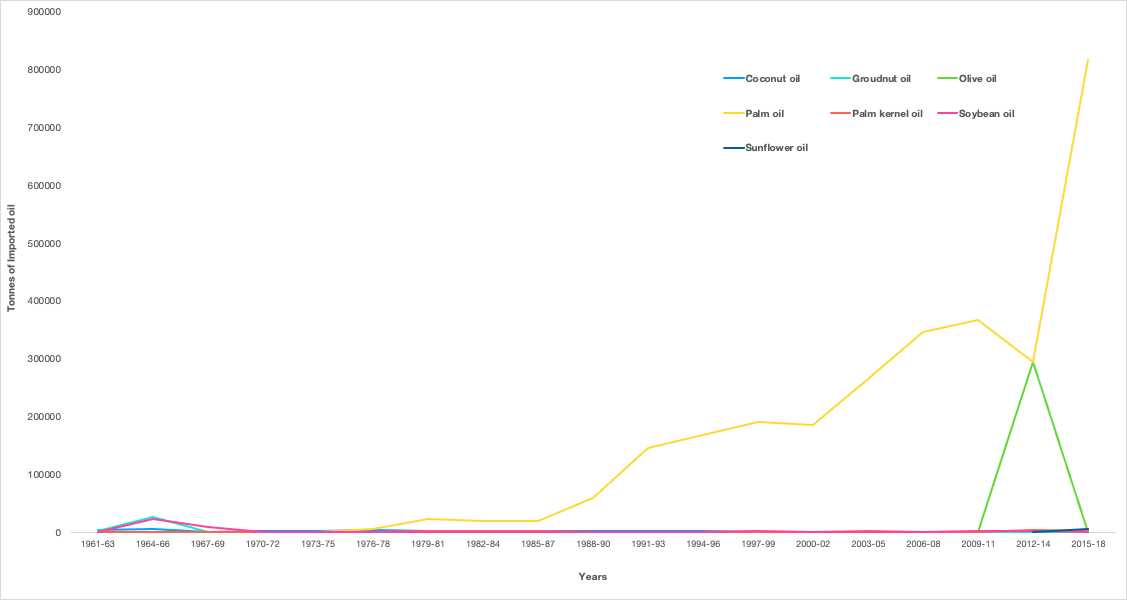


**Supplemental Figure 2**. An overview of edible oil imports from 1961-2018 in Myanmar


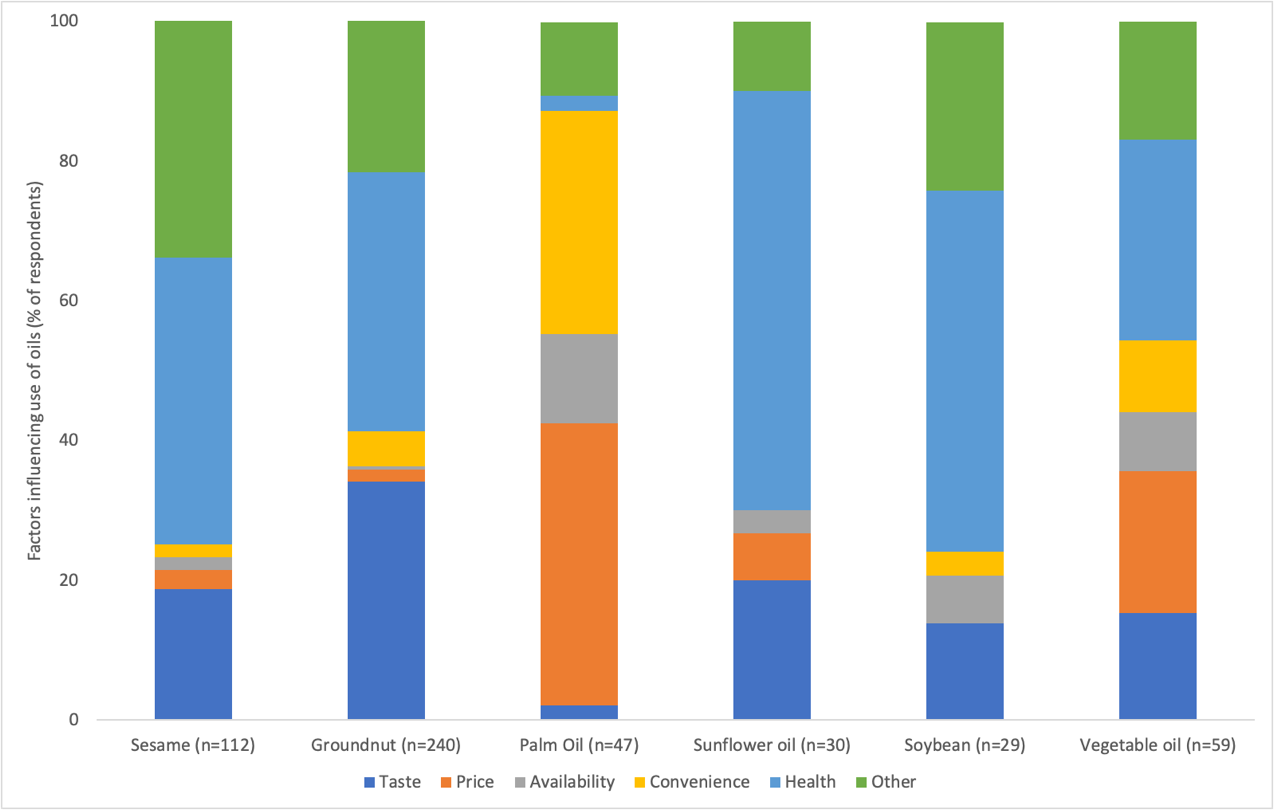


**Supplemental Figure 3.** The main factors influencing the use of different oil types among consumers

**Supplemental Table 3.** Socio-demographic characteristics of study participants (n=362*)

| Demographic Characteristic | | % of Sample |
| --- | --- | --- |
| Sex (%) | Male | 49.2% |
|  | Female | 50.8% |
| Age (mean ± SD) |  | 40.8 ± 12 |
| Ethnicity (%) | Burma | 80.4% |
|  | Karen | 5.8% |
|  | Indian | 5.5% |
|  | Daweil | 3% |
|  | Rakhine | 3% |
|  | Other | 2.3% |
| Highest level of education completed (%) | Less than primary school | 14.9% |
|  | Primary School | 30.1% |
|  | Secondary School | 30.4% |
|  | High School | 5.4% |
|  | College/University | 19.1% |
| Employment type (%) | Government employee | 4.7% |
|  | Non-government employee | 14.6% |
|  | Self-employed | 69.8% |
|  | Homemaker | 8% |
|  | Retired | 1.4% |
|  | Other | 1.5% |

*Please note: we removed 38 respondents from the study sample given that they did not reside in the study setting
